# Vegetarian and vegan diets and risks of total and site-specific fractures: results from the prospective EPIC-Oxford study

**DOI:** 10.1186/s12916-020-01815-3

**Published:** 2020-11-23

**Authors:** Tammy Y. N. Tong, Paul N. Appleby, Miranda E. G. Armstrong, Georgina K. Fensom, Anika Knuppel, Keren Papier, Aurora Perez-Cornago, Ruth C. Travis, Timothy J. Key

**Affiliations:** 1 Cancer Epidemiology Unit, Nuffield Department of Population Health, University of Oxford, Richard Doll Building, Old Road Campus, Oxford, OX3 7LF UK; 2 Centre for Exercise, Nutrition and Health Sciences, School for Policy Studies, University of Bristol, Bristol, UK

**Keywords:** Vegetarian diets, Vegan diets, Fractures, Bone health, Calcium, Protein, Body mass index, Prospective study

## Abstract

**Background:**

There is limited prospective evidence on possible differences in fracture risks between vegetarians, vegans, and non-vegetarians. We aimed to study this in a prospective cohort with a large proportion of non-meat eaters.

**Methods:**

In EPIC-Oxford, dietary information was collected at baseline (1993–2001) and at follow-up (≈ 2010). Participants were categorised into four diet groups at both time points (with 29,380 meat eaters, 8037 fish eaters, 15,499 vegetarians, and 1982 vegans at baseline in analyses of total fractures). Outcomes were identified through linkage to hospital records or death certificates until mid-2016. Using multivariable Cox regression, we estimated the risks of total (*n* = 3941) and site-specific fractures (arm, *n* = 566; wrist, *n* = 889; hip, *n* = 945; leg, *n* = 366; ankle, *n* = 520; other main sites, i.e. clavicle, rib, and vertebra, *n* = 467) by diet group over an average of 17.6 years of follow-up.

**Results:**

Compared with meat eaters and after adjustment for socio-economic factors, lifestyle confounders, and body mass index (BMI), the risks of hip fracture were higher in fish eaters (hazard ratio 1.26; 95% CI 1.02–1.54), vegetarians (1.25; 1.04–1.50), and vegans (2.31; 1.66–3.22), equivalent to rate differences of 2.9 (0.6–5.7), 2.9 (0.9–5.2), and 14.9 (7.9–24.5) more cases for every 1000 people over 10 years, respectively. The vegans also had higher risks of total (1.43; 1.20–1.70), leg (2.05; 1.23–3.41), and other main site fractures (1.59; 1.02–2.50) than meat eaters. Overall, the significant associations appeared to be stronger without adjustment for BMI and were slightly attenuated but remained significant with additional adjustment for dietary calcium and/or total protein. No significant differences were observed in risks of wrist or ankle fractures by diet group with or without BMI adjustment, nor for arm fractures after BMI adjustment.

**Conclusions:**

Non-meat eaters, especially vegans, had higher risks of either total or some site-specific fractures, particularly hip fractures. This is the first prospective study of diet group with both total and multiple specific fracture sites in vegetarians and vegans, and the findings suggest that bone health in vegans requires further research.

## Background

Fractures in adulthood and older ages are a common occurrence which pose a significant burden to health systems worldwide [1]. Previous epidemiological studies have shown that vegetarians had lower bone mineral density (BMD) than non-vegetarians [2, 3], but the associations of vegetarian diets with fracture risks are unclear. Potential risk differences are plausible however, owing to differences in several dietary factors, such as the substantially lower intakes of calcium in vegans [4, 5], lower intakes of dietary protein in both vegetarians and vegans [6, 7], and the lower body mass index (BMI) of non-meat eaters [2, 8].

Prior studies have linked both calcium and protein intakes to bone health, but their relationships with fracture risks are nuanced. For calcium, although previous meta-analyses have found that calcium supplements are effective in producing small increases in BMD [9], it is less clear whether this degree of improvement would be sufficient to reduce fracture risks [10]. However, a recent meta-analysis of randomised trials showed that combined vitamin D and calcium supplementation, but not vitamin D supplementation alone, was effective in fracture prevention, therefore supporting the importance of calcium [11]. For protein, while older studies suggested that high protein intake might lead to higher calcium excretion and therefore weaker bones [12], more recent evidence has suggested a positive association between protein and bone health, although this might not translate to differences in fracture risk [13]. In addition, BMI is also an important factor for fracture risk [14], and a recent study suggested that the lower BMD observed in US vegetarians might be largely explained by their lower BMI and waist circumference [15]. However, the directions of association between BMI and fracture risk differ across fracture sites, and low BMI has been associated with a higher risk of hip fracture but lower risk of ankle fracture [14].

The largest study to date on vegetarian diet group and fracture risks came from previous analyses in EPIC-Oxford on around 30,000 participants, and reported that vegans, but not vegetarians, had higher risks of total fractures, although this analysis had a short follow-up (5 years) and relied on self-reported outcome data [16]. The only two other studies on the topic included a small number of participants and did not report on site-specific fractures [17, 18]. Hence, the possible differences in fracture risks by vegetarian diet groups are still unclear, and it is not known whether the risks might differ by fracture sites.

Therefore, the aim of this study was to examine the risks of total and site-specific fractures in a prospective cohort with close to 18 years of average follow-up, including a large proportion of non-meat eaters, and with outcome data based on record linkage.

## Methods

### Study population

EPIC-Oxford is a prospective cohort study which recruited approximately 65,000 men and women across the UK between 1993 and 2001, via either general practices or by postal questionnaire. Details of the recruitment process and eligibility criteria for inclusion in the analyses can be found in Additional File 1: Supplementary methods [4, 19] and in the participant flow chart (Additional File 1: Fig. S1). The study has approval by a Multicentre Research Ethics Committee (Scotland A Research Ethics Committee). All participants provided written informed consent.

### Classification of diet group

At recruitment, participants completed a questionnaire which asked about diet, socio-demographic characteristics, lifestyle, and medical history. A follow-up questionnaire which asked similar questions was sent to participants in 2010. Based on the responses to both questionnaires (if the participant completed the follow-up questionnaire), the participants were categorised into meat eaters, fish eaters (did not eat meat but ate fish), vegetarians (did not eat meat or fish, but ate one or both of dairy or eggs), and vegans (participants who did not eat meat, fish, dairy, and eggs) at both time points. Further details on the questionnaires, classification of diet group including agreement of diet group at baseline and follow-up, and data collection of other baseline characteristics can be found in Additional File 1: Supplementary methods [20–27].

### Outcome assessment

Participants were followed up for health outcomes via record linkage to National Health Service records until 31 March 2016 in England, 31 May 2016 in Wales, and 31 October 2016 in Scotland. The outcomes of interest were the first recorded hospital admission (inpatient admissions in England, inpatient admissions and day cases in Wales and Scotland) or death from total and site-specific fractures, including fractures of the arm (i.e. humerus, radius, and ulna), wrist, hip, leg (i.e. femur [excluding neck of femur], patella, tibia, and fibula), ankle, and other main sites (i.e. clavicle, rib, or vertebra), identified by the relevant 9th or 10th revisions of the World Health Organization’s International Classification of Diseases (ICD-9/ICD-10) codes (Additional File 1: Table S1). For total fractures, incidence was defined as the first recorded occurrence of any diagnosis of any fracture; for site-specific fractures, incidence was defined as the first recorded occurrence of any fracture at that particular site, without censoring for previous fractures at other sites. Fractures at the clavicle, rib, and vertebra were examined as one composite outcome due to the small number of cases at these sites, but the three sites were examined separately in secondary analyses.

### Statistical analyses

Baseline characteristics and food and nutrient intakes of the EPIC-Oxford participants were summarised by diet group. Cox proportional hazards regression models were used to estimate the hazard ratios (HRs) and 95% confidence intervals (CIs) for the associations between the four diet groups (meat eaters, fish eaters, vegetarians, vegans) and total and each site-specific fracture of interest, using meat eaters as the reference group. The underlying time variable was the age at recruitment to the age at diagnosis, death, or administrative censoring, whichever occurred first. For participants who completed both the baseline and follow-up questionnaires, diet group and relevant time-varying covariates (smoking and alcohol consumption, BMI, dietary calcium or protein) were updated at follow-up; otherwise, the baseline dietary or covariate information was carried forward.

All analyses were stratified by sex, method of recruitment, and region of residence, and adjusted for year of recruitment, ethnicity, Townsend deprivation index [25], education level, physical activity [26], smoking, alcohol consumption, dietary supplement use, height, and in women menopausal status, hormone replacement therapy use, and parity. We tested models with and without adjustment for BMI. Details on the categorisation of covariates can be found in the Supplementary methods. The proportional hazards assumption was assessed on the basis of Schoenfeld residuals and was not violated for the variables of interest in the adjusted model for any of the outcomes. Subsequently, we estimated absolute rate differences based on the BMI adjusted model, using a previously reported method [28].

To evaluate the influence of dietary calcium and protein on the associations, we included models further adjusting for either dietary calcium or dietary protein intake, and simultaneously adjusting for both variables. Additional analyses were also performed limited to people with sufficient dietary calcium (≥ 700 mg/day) or dietary protein intake (≥ 0.75 g of protein per day/kg body weight) in accordance with UK dietary guidelines [29, 30].

As sensitivity analyses, we repeated the analyses (with adjustment for BMI) further adjusting for energy intake, excluding the first 5 years of follow-up, excluding participants with prior diseases (baseline history of diabetes, heart disease, stroke, or cancer), excluding participants who were receiving long-term treatment for any illness, and with multiple imputation for missing covariates [31]. Heterogeneity of results by age at recruitment (below and above age 50), sex, menopausal status, physical activity level (inactive/low and moderate/high activity), and BMI (below and above 22.5 kg/m^2^) was assessed for total and hip fractures, which had the largest numbers of cases. Cut-offs of age and BMI were chosen to ensure a reasonable distribution of number of cases in categories across all diet groups, based on analyses of total fractures.

All analyses were performed using Stata version 15.1 (StataCorp, TX, USA), and 2-sided *p* values < 0.05 were considered significant. The forest plot was generated using R (R Foundation for Statistical Computing, Vienna, Austria).

## Results

The study population included a minimum of 54,898 participants (in analyses for total fractures), of whom 30,391 had repeated measures of diet 14 years later (details in Additional File 1: Fig. S1). Baseline characteristics in the overall cohort are tabulated by the four diet groups in Table 1, and separately for men and women in Additional File 1: Table S2. Other dietary and nutrient intakes are tabulated by the four diet groups, separately for men and women in Additional File 1: Table S3. A summary description of the baseline and dietary characteristics can be found in Additional File 1: Supplementary results.

**Table 1:** Baseline characteristics of EPIC-Oxford participants by diet group

	Diet group			
Characteristics, mean (SD) or ***n*** (%)	Meat eaters (***n*** = 29,380)	Fish eaters (***n*** = 8037)	Vegetarians (***n*** = 15,499)	Vegans (***n*** = 1982)
**Socio-demographic**				
Age, years (SD)	50.1 (13.1)	42.7 (13.3)	40.0 (13.5)	38.9 (13.6)
Sex, women (%)	22,591 (76.9)	6614 (82.3)	11,911 (76.9)	1266 (63.9)
Top socio-economic quartile (%)^a^	6947 (27.4)	1534 (21.9)	2952 (21.7)	308 (17.6)
Higher education (%)	8198 (30.8)	3457 (45.3)	6289 (42.7)	797 (42.8)
White ethnicity (%)	28,334 (98.6)	7717 (97.8)	14,764 (97.4)	1848 (97.0)
**Lifestyle**				
Current smokers (%)	3623 (12.4)	805 (10.1)	1554 (10.1)	212 (10.8)
Alcohol consumption, g/day (SD)	9.7 (12.6)	9.8 (12.2)	9.2 (12.6)	8.3 (13.5)
Moderate or high physical activity (%)	7516 (30.7)	2795 (39.4)	5400 (39.0)	807 (45.3)
Dietary supplement use (%)^b^	15,941 (55.4)	5046 (64.3)	8660 (56.9)	1008 (51.9)
**Health characteristics and medical history**				
Body mass index, kg/m^2^ (SD)	24.5 (4.0)	23.0 (3.4)	22.9 (3.5)	22.1 (3.0)
< 20 kg/m^2^ (%)	2365 (8.3)	1178 (15.1)	2508 (16.8)	456 (23.8)
≥ 25 kg/m^2^ (%)	10,407 (36.6)	1602 (20.6)	3082 (20.7)	268 (14.0)
Height in men, cm (SD)	177.6 (7.0)	178.1 (6.7)	178.1 (7.0)	178.1 (6.9)
Height in women, cm (SD)	163.7 (6.7)	164.6 (6.8)	164.4 (6.8)	164.5 (6.9)
Premenopausal (%)^c^	9625 (44.0)	4410 (68.2)	8856 (75.7)	940 (75.6)
Postmenopausal (%)^c^	10,106 (46.2)	1570 (24.3)	2107 (18.0)	235 (18.9)
Hormone replacement therapy use (%)^c^	5945 (26.7)	805 (12.3)	992 (8.4)	69 (5.6)
One or more children (%)^c^	16,856 (75.2)	3524 (53.8)	5215 (44.3)	431 (34.4)
**Dietary information**				
Energy, kJ/day (SD)	8286 (2246)	7940 (2208)	7866 (2213)	7342 (2315)
Dietary calcium, mg/day (SD)	1005 (314)	1033 (349)	1030 (369)	591 (237)
Protein, % energy (SD)	17.0 (3.0)	14.7 (2.4)	13.6 (2.1)	13.3 (2.3)

Estimates shown are mean (SD) or numbers (%), as stated in left column. Percentages were estimated excluding participants with missing responses

^a^Based on Townsend deprivation index

^b^Defined as regularly taking any vitamins, minerals, fish oils, fibre, or other food supplements during the last 12 months

^c^In women only

**Fig. 1:**
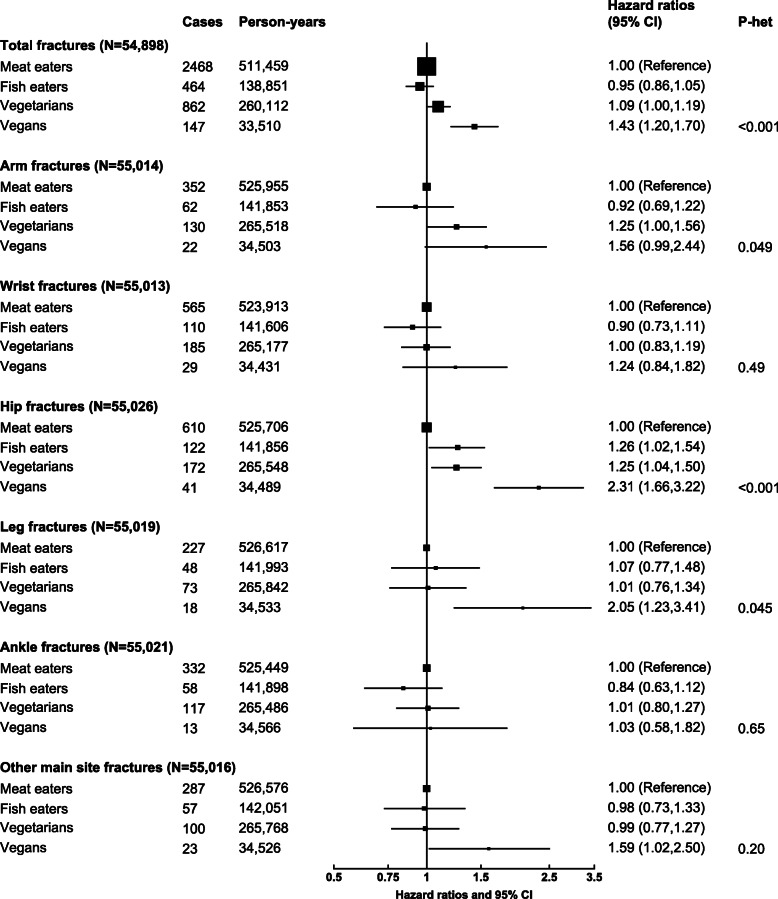
Risks of total and site-specific fractures by diet group in EPIC-Oxford. Estimates also shown in Table 2 as model 2. All analyses were stratified by sex, method of recruitment (general practice or postal), and region (7 categories), and adjusted for year of recruitment (per year from ≤ 1994 to ≥ 1999), ethnicity (white, other, unknown), Townsend deprivation index (quartiles, unknown), education level (no qualifications, basic secondary (e.g. O level), higher secondary (e.g. A level), degree, unknown), physical activity (inactive, low activity, moderately active, very active, unknown), smoking (never, former, light, heavy, unknown), alcohol consumption (< 1 g, 1–7 g, 8–15 g, 16+ g/day), dietary supplement use (no, yes, unknown), height (5 cm categories from < 155 to ≥ 185 cm, unknown), body mass index (< 18.5, 18.5–19.9, 20–22.4, 22.5–24.9, 25–27.4, 27.5–29.9, 30–32.4, ≥ 32.5 kg/m^2^, unknown), and in women menopausal status (premenopausal, perimenopausal, postmenopausal, unknown), hormone replacement therapy use (never, ever, unknown), and parity (none, 1–2, ≥ 3, unknown). Other main site fractures are defined as fractures of the clavicle, rib, or vertebra

Over an average of 17.6 years of follow-up, we observed 3941 cases of total fractures (including 12 first reported at death; 943,934 person-years), 566 arm fractures (1 at death; 967,829 person-years), 889 wrist fractures (965,127 person-years), 945 hip fractures (1 at death; 967,599 person-years), 366 leg fractures (1 at death; 968,985 person-years), 520 ankle fractures (967,399 person-years), and 467 other main site fractures (968,921 person-years). The results of longitudinal associations between diet group and total and site-specific fractures are shown in Fig. 1 and Table 2. Absolute rate differences (AD) in the outcomes by diet group based on the BMI adjusted model are shown in Table 3.

**Table 2:** Risks of total and site-specific fractures by diet group in EPIC-Oxford, with varying levels of adjustments

Fracture site/diet group	***N*** cases	Person-years	Age at event^a^	Hazard ratios (95% confidence intervals)				
				Model 1^b^	Model 2^c^	Model 3^d^	Model 4^e^	Model 5^f^
**Total fractures (*N*** **= 54,898)**								
Meat eaters	2468	511,459	68.5 (13.1)	Reference	Reference	Reference	Reference	Reference
Fish eaters	464	138,851	64.3 (14.5)	0.97 (0.87, 1.07)	0.95 (0.86, 1.05)	0.95 (0.85, 1.05)	0.94 (0.84, 1.04)	0.94 (0.84, 1.05)
Vegetarians	862	260,112	60.7 (16.4)	1.11 (1.02, 1.21)	1.09 (1.00, 1.19)	1.08 (1.00, 1.18)	1.07 (0.97, 1.17)	1.07 (0.97, 1.18)
Vegans	147	33,510	59.9 (17.4)	1.50 (1.26, 1.78)	1.43 (1.20, 1.70)	1.31 (1.10, 1.57)	1.39 (1.16, 1.67)	1.30 (1.08, 1.56)
*p*-heterogeneity^g^				< 0.001	< 0.001	0.003	0.001	0.009
**Arm fractures (*N*** **= 55,014)**								
Meat eaters	352	525,955	69.9 (12.4)	Reference	Reference	Reference	Reference	Reference
Fish eaters	62	141,853	66.1 (13.0)	0.93 (0.71, 1.24)	0.92 (0.69, 1.22)	0.92 (0.69, 1.22)	0.85 (0.64, 1.13)	0.85 (0.64, 1.13)
Vegetarians	130	265,518	63.7 (15.4)	1.28 (1.03, 1.60)	1.25 (1.00, 1.56)	1.25 (1.00, 1.56)	1.12 (0.87, 1.43)	1.12 (0.87, 1.43)
Vegans	22	34,503	58.0 (16.1)	1.67 (1.07, 2.61)	1.56 (0.99, 2.44)	1.54 (0.96, 2.46)	1.39 (0.87, 2.22)	1.39 (0.86, 2.25)
*p*-heterogeneity^g^				0.020	0.049	0.056	0.19	0.20
**Wrist fractures (*N*** **= 55,013)**								
Meat eaters	565	523,913	67.3 (11.4)	Reference	Reference	Reference	Reference	Reference
Fish eaters	110	141,606	64.4 (13.6)	0.93 (0.75, 1.15)	0.90 (0.73, 1.11)	0.90 (0.73, 1.12)	0.92 (0.74, 1.15)	0.94 (0.75, 1.17)
Vegetarians	185	265,177	60.2 (14.4)	1.03 (0.86, 1.24)	1.00 (0.83, 1.19)	1.00 (0.83, 1.20)	1.03 (0.84, 1.26)	1.05 (0.86, 1.29)
Vegans	29	34,431	58.8 (16.2)	1.33 (0.91, 1.96)	1.24 (0.84, 1.82)	1.14 (0.76, 1.70)	1.28 (0.86, 1.90)	1.19 (0.79, 1.79)
*p*-heterogeneity^g^				0.39	0.49	0.70	0.49	0.67
**Hip fractures (*N*** **= 55,026)**								
Meat eaters	610	525,706	76.8 (9.5)	Reference	Reference	Reference	Reference	Reference
Fish eaters	122	141,856	74.6 (11.4)	1.33 (1.08, 1.62)	1.26 (1.02, 1.54)	1.26 (1.03, 1.54)	1.24 (1.01, 1.53)	1.25 (1.01, 1.55)
Vegetarians	172	265,548	74.5 (11.9)	1.34 (1.12, 1.61)	1.25 (1.04, 1.50)	1.24 (1.03, 1.49)	1.20 (0.98, 1.47)	1.21 (0.99, 1.49)
Vegans	41	34,489	74.1 (13.3)	2.64 (1.90, 3.67)	2.31 (1.66, 3.22)	1.97 (1.39, 2.79)	2.21 (1.56, 3.12)	1.94 (1.35, 2.77)
*p*-heterogeneity^g^				< 0.001	< 0.001	< 0.001	< 0.001	0.002
**Leg fractures (*N*** **= 55,019)**								
Meat eaters	227	526,617	68.7 (12.3)	Reference	Reference	Reference	Reference	Reference
Fish eaters	48	141,993	62.8 (13.8)	1.06 (0.77, 1.47)	1.07 (0.77, 1.48)	1.06 (0.77, 1.47)	1.06 (0.76, 1.49)	1.05 (0.75, 1.47)
Vegetarians	73	265,842	61.3 (16.0)	1.00 (0.75, 1.33)	1.01 (0.76, 1.34)	1.00 (0.75, 1.33)	0.99 (0.72, 1.37)	0.99 (0.72, 1.36)
Vegans	18	34,533	59.1 (19.3)	2.04 (1.23, 3.38)	2.05 (1.23, 3.41)	1.83 (1.07, 3.14)	2.02 (1.19, 3.44)	1.81 (1.04, 3.15)
*p*-heterogeneity^g^				0.044	0.045	0.16	0.053	0.17
**Ankle fractures (*N*** **= 55,021)**								
Meat eaters	332	525,449	63.0 (12.1)	Reference	Reference	Reference	Reference	Reference
Fish eaters	58	141,898	56.0 (10.2)	0.79 (0.59, 1.05)	0.84 (0.63, 1.12)	0.84 (0.63, 1.12)	0.88 (0.65, 1.19)	0.89 (0.66, 1.20)
Vegetarians	117	265,486	54.7 (14.4)	0.94 (0.75, 1.19)	1.01 (0.80, 1.27)	1.01 (0.80, 1.27)	1.10 (0.85, 1.43)	1.11 (0.85, 1.44)
Vegans	13	34,566	51.1 (14.8)	0.90 (0.51, 1.58)	1.03 (0.58, 1.82)	0.91 (0.50, 1.63)	1.13 (0.63, 2.03)	1.00 (0.55, 1.82)
*p*-heterogeneity^g^				0.44	0.65	0.65	0.59	0.60
**Other main site fractures**^h^ **(*N*** **= 55,016)**								
Meat eaters	287	526,576	69.1 (14.1)	Reference	Reference	Reference	Reference	Reference
Fish eaters	57	142,051	63.1 (15.1)	1.01 (0.75, 1.36)	0.98 (0.73, 1.33)	0.98 (0.72, 1.32)	0.98 (0.72, 1.33)	0.96 (0.71, 1.31)
Vegetarians	100	265,768	59.4 (16.7)	1.03 (0.80, 1.32)	0.99 (0.77, 1.27)	0.98 (0.76, 1.26)	0.97 (0.73, 1.28)	0.95 (0.72, 1.26)
Vegans	23	34,526	58.2 (16.2)	1.73 (1.11, 2.70)	1.59 (1.02, 2.50)	1.66 (1.03, 2.67)	1.55 (0.97, 2.48)	1.61 (0.98, 2.62)
*p*-heterogeneity^g^				0.11	0.20	0.18	0.23	0.20

^a^In cases only, as mean years (SD)

^b^Model 1 was stratified by sex, method of recruitment (general practice or postal), and region (7 categories), and adjusted for year of recruitment (per year from ≤ 1994 to ≥ 1999), ethnicity (white, other, unknown), Townsend deprivation index (quartiles, unknown), education level (no qualifications, basic secondary (e.g. O level), higher secondary (e.g. A level), degree, unknown), physical activity (inactive, low activity, moderately active, very active, unknown), smoking (never, former, light, heavy, unknown), alcohol consumption (< 1 g, 1–7 g, 8–15 g, 16+ g/day), dietary supplement use (no, yes, unknown), height (5 cm categories from < 155 to ≥ 185 cm, unknown), and in women menopausal status (premenopausal, perimenopausal, postmenopausal, unknown), hormone replacement therapy use (never, ever, unknown), and parity (none, 1–2, ≥ 3, unknown)

^c^Model 2 was model 1 plus BMI (< 18.5, 18.5–19.9, 20–22.4, 22.5–24.9, 25–27.4, 27.5–29.9, 30–32.4, ≥ 32.5 kg/m^2^, unknown)

^d^Model 3 was model 2 plus dietary calcium intake (< 525, 525–699, 700–899, 900–1199, ≥ 1200 mg)

^e^Model 4 was model 2 plus protein intake (< 13, 13–14.4, 14.5–15.9, 16–18.0, ≥ 18.1 of % energy)

^f^Model 5 was model 2 plus dietary calcium intake (< 525, 525–699, 700–899, 900–1199, ≥ 1200 mg) and protein intake (< 13, 13–14.4, 14.5–15.9, 16–18.0, ≥ 18.1 of % energy)

^g^*p*-heterogeneity represents heterogeneity in risk between diet group based on Wald’s tests

^h^Defined as fractures of the clavicle, rib, or vertebra

**Table 3:** Absolute rate differences of total and site-specific fractures by diet group in EPIC-Oxford

Fracture site/diet group	Predicted incidence^a^		Absolute rate difference^b^	
	Per 1000 person-years	Per 1000 people over 10 years	Per 1000 person-years	Per 1000 people over 10 years
**Total fractures**				
Meat eaters	4.8 (4.6, 5.1)	47.2 (44.9, 49.7)	Reference	Reference
Fish eaters	4.6 (4.2, 5.0)	44.8 (41.0, 49.0)	− 0.2 (− 0.6, 0.2)	− 2.4 (− 6.2, 1.8)
Vegetarians	5.3 (4.9, 5.6)	51.3 (48.0, 54.8)	0.4 (0.1, 0.8)	4.1 (0.8, 7.6)
Vegans	6.9 (5.8, 8.1)	66.6 (56.8, 78.1)	2.0 (1.0, 3.3)	19.4 (9.6, 30.9)
**Total fractures in men**				
Meat eaters	3.9 (3.4, 4.4)	37.9 (33.4, 42.8)	Reference	Reference
Fish eaters	3.6 (2.9, 4.5)	35.5 (28.5, 44.3)	− 0.2 (− 1.0, 0.7)	− 2.3 (− 9.4, 6.4)
Vegetarians	4.1 (3.5, 4.7)	40.0 (34.9, 45.8)	0.2 (− 0.3, 0.8)	2.1 (− 3.0, 7.9)
Vegans	4.5 (3.4, 6.1)	44.3 (33.2, 59.1)	0.7 (− 0.5, 2.2)	6.5 (− 4.7, 21.3)
**Total fractures in women**				
Meat eaters	5.1 (4.8, 5.4)	49.9 (47.2, 52.7)	Reference	Reference
Fish eaters	4.9 (4.4, 5.4)	47.5 (43.1, 52.3)	− 0.3 (− 0.7, 0.3)	− 2.4 (− 6.8, 2.4)
Vegetarians	5.6 (5.2, 6.0)	54.5 (50.5, 58.8)	0.5 (0.1, 0.9)	4.6 (0.6, 8.9)
Vegans	7.8 (6.4, 9.5)	75.1 (62.0, 90.9)	2.7 (1.3, 4.4)	25.2 (12.1, 41.0)
**Arm fractures**				
Meat eaters	0.7 (0.6, 0.8)	6.7 (5.8, 7.6)	Reference	Reference
Fish eaters	0.6 (0.5, 0.8)	6.1 (4.8, 7.9)	− 0.1 (− 0.2, 0.1)	− 0.5 (− 1.9, 1.2)
Vegetarians	0.8 (0.7, 1.0)	8.3 (7.0, 9.9)	0.2 (0.0, 0.3)	1.6 (0.3, 3.2)
Vegans	1.0 (0.7, 1.6)	10.4 (6.8, 15.8)	0.4 (0.0, 0.9)	3.7 (0.1, 9.2)
**Wrist fractures**				
Meat eaters	1.1 (1.0, 1.2)	10.7 (9.7, 11.9)	Reference	Reference
Fish eaters	1.0 (0.8, 1.2)	9.7 (8.0, 11.6)	− 0.1 (− 0.3, 0.1)	− 1.1 (− 2.7, 0.9)
Vegetarians	1.1 (0.9, 1.2)	10.7 (9.2, 12.4)	0.0 (− 0.1, 0.2)	0.0 (− 1.5, 1.7)
Vegans	1.3 (0.9, 1.9)	13.2 (9.2, 19.1)	0.3 (− 0.2, 0.8)	2.5 (− 1.6, 8.4)
**Hip fractures**				
Meat eaters	1.2 (1.0, 1.3)	11.5 (10.4, 12.8)	Reference	Reference
Fish eaters	1.5 (1.2, 1.7)	14.5 (12.1, 17.3)	0.3 (0.1, 0.6)	2.9 (0.6, 5.7)
Vegetarians	1.5 (1.2, 1.7)	14.4 (12.4, 16.8)	0.3 (0.1, 0.5)	2.9 (0.9, 5.2)
Vegans	2.7 (2.0, 3.7)	26.5 (19.4, 36.0)	1.5 (0.8, 2.5)	14.9 (7.9, 24.5)
**Leg fractures**				
Meat eaters	0.4 (0.4, 0.5)	4.3 (3.6, 5.1)	Reference	Reference
Fish eaters	0.5 (0.3, 0.6)	4.6 (3.5, 6.1)	0.0 (− 0.1, 0.2)	0.3 (− 0.8, 1.8)
Vegetarians	0.4 (0.3, 0.5)	4.3 (3.4, 5.5)	0.0 (− 0.1, 0.1)	0.0 (− 0.9, 1.2)
Vegans	0.9 (0.5, 1.4)	8.8 (5.5, 14.1)	0.5 (0.1, 1.0)	4.5 (1.2, 9.8)
**Ankle fractures**				
Meat eaters	0.6 (0.5, 0.7)	6.3 (5.5, 7.2)	Reference	Reference
Fish eaters	0.5 (0.4, 0.7)	5.3 (4.1, 6.8)	− 0.1 (− 0.2, 0.1)	− 1.0 (− 2.2, 0.5)
Vegetarians	0.6 (0.5, 0.8)	6.4 (5.3, 7.7)	0.0 (− 0.1, 0.1)	0.1 (− 1.0, 1.4)
Vegans	0.7 (0.4, 1.1)	6.5 (3.7, 11.2)	0.0 (− 0.3, 0.5)	0.2 (− 2.6, 4.9)
**Other main site fractures**^c^				
Meat eaters	0.5 (0.5, 0.6)	5.4 (4.7, 6.3)	Reference	Reference
Fish eaters	0.5 (0.4, 0.7)	5.4 (4.1, 6.9)	0.0 (− 0.1, 0.1)	− 0.1 (− 1.3, 1.5)
Vegetarians	0.5 (0.4, 0.7)	5.4 (4.4, 6.5)	0.0 (− 0.1, 0.1)	− 0.1 (− 1.0, 1.1)
Vegans	0.9 (0.6, 1.3)	8.7 (5.7, 13.1)	0.3 (0.0, 0.8)	3.2 (0.3, 7.7)

^a^For meat eaters, calculated as (1 − *S*_r_) × 1000, where *S*_r_ = (1 − observed incidence in meat eaters), representing the average survival (or non-incidence) rate in the meat eaters, or *S*_r_ = (1 − observed incidence in meat eaters)^10^, representing the predicted 10-year survival rate in the meat eaters. For all other diet groups, calculated as (1 − *S*_r_^HR^) × 1000, where HR represents the hazard ratio or confidence intervals of the hazard ratio for each outcome in that diet group, and *S*_r_^HR^ represents either the predicted average survival or the predicted 10-year survival rate in the diet group, as indicated in the column heading. Hazard ratios and confidence intervals were based on covariate adjustment as listed for Table 2 model 2 or Table 5 (for total fractures in men and women), and expressed as floating absolute risks

^b^Calculated as the difference between the predicted incidence per 1000 person-years or per 1000 people over 10 years between each diet group and the meat eaters

^c^Defined as fractures of the clavicle, rib, or vertebra

Compared with meat eaters, vegetarians (HR 1.11; 95% CI 1.02, 1.21) and vegans (1.50; 1.26, 1.78) had higher risks of total fractures after adjustment for confounders (Table 2 model 1). The associations attenuated with additional adjustment of BMI (vegetarians—1.09; 1.00, 1.19; vegans—1.43; 1.20, 1.70), but remained clearly significant in vegans (Table 2 model 2, Fig. 1). The equivalent rate differences were 4.1 (0.8, 7.6) more cases in vegetarians and 19.4 (9.6, 30.9) more cases in vegans for every 1000 people over 10 years. The associations were attenuated further but remained significant in vegans with additional adjustment for dietary calcium (1.31; 1.10, 1.57, Table 2 model 3), total dietary protein (1.39; 1.16, 1.67, Table 2 model 4), or both dietary factors simultaneously (1.30; 1.08, 1.56, Table 2 model 5).

For site-specific fractures (Fig. 1 and Table 2), the largest magnitudes in risk difference by diet group were observed for hip fractures. After adjustment for BMI, the risks were higher in fish eaters (HR 1.26; 1.02, 1.54, or AD 2.9; 0.6, 5.7), vegetarians (HR 1.25; 1.04, 1.50, or AD 2.9; 0.9, 5.2), and vegans (HR 2.31; 1.66, 3.22, or AD 14.9; 7.9, 24.5) than meat eaters. Similar to the findings for total fractures, the associations appeared stronger before BMI adjustment and attenuated but remained strongly significant in vegans after further adjustment for both calcium and protein.

For the other sites, after adjustment for BMI, the vegans had a higher risk of leg fractures (2.05; 1.23, 3.41) and other main site fractures (clavicle, rib, vertebra, 1.59; 1.02, 2.50) than the meat eaters (Fig. 1 and Table 2). When the other main site fractures were examined separately, a significantly higher risk was observed in the vegans for vertebral fracture (2.42; 2.31, 4.48), but not for the other two sites (Additional File 1: Table S4). No significant differences in risks between diet groups were observed for arm, wrist, or ankle fracture, after adjustment for BMI (Fig. 1 and Table 2), although a higher risk of arm fractures was observed in both vegetarians (1.28; 1.03, 1.60) and vegans (1.67; 1.07, 2.61) in the multivariable model before BMI adjustment (Table 2 model 1).

Results from secondary analyses are reported in more detail in the Supplementary results. Overall, results were consistent when the analyses were restricted to participants with sufficient intakes of calcium and protein (Table 4), and also in other secondary analyses, including with further adjustment for energy intake, excluding the first 5 years of follow-up, excluding participants with prior diseases or receiving long-term treatment for any illness, or with multiple imputation for missing covariates (Additional File 1: Table S5).

**Table 4:** Risks of total and site-specific fractures by diet group in EPIC-Oxford, in participants with adequate levels of dietary calcium or protein

Fracture site/diet group	Hazard ratios (95% confidence intervals)^a^					
	***N*** cases	Dietary calcium ≥ 700 mg/day	***N*** cases	Dietary protein ≥ 0.75 g per day/kg body weight	***N*** cases	Dietary calcium ≥ 700 mg/day plus dietary protein ≥ 0.75 g per day/kg body weight
**Total fractures**						
Meat eaters	2077	Reference	2188	Reference	1925	Reference
Fish eaters	377	0.92 (0.82, 1.03)	376	0.92 (0.82, 1.03)	332	0.90 (0.80, 1.02)
Vegetarians	700	1.08 (0.98, 1.19)	648	1.07 (0.97, 1.18)	583	1.06 (0.96, 1.18)
Vegans	49	1.50 (1.12, 1.99)	103	1.52 (1.24, 1.87)	44	1.45 (1.07, 1.97)
*p*-heterogeneity^b^		0.003		< 0.001		0.009
**Arm fractures**						
Meat eaters	300	Reference	304	Reference	273	Reference
Fish eaters	54	0.96 (0.71, 1.30)	49	0.89 (0.65, 1.21)	46	0.91 (0.66, 1.26)
Vegetarians	107	1.27 (0.99, 1.62)	98	1.24 (0.97, 1.59)	90	1.25 (0.96, 1.62)
Vegans	8	1.76 (0.86, 3.61)	15	1.58 (0.92, 2.70)	7	1.61 (0.75, 3.46)
*p*-heterogeneity^b^		0.10		0.091		0.18
**Wrist fractures**						
Meat eaters	475	Reference	503	Reference	436	Reference
Fish eaters	89	0.88 (0.70, 1.11)	96	0.93 (0.74, 1.16)	82	0.91 (0.71, 1.16)
Vegetarians	146	0.96 (0.79, 1.18)	140	0.97 (0.79, 1.19)	126	0.99 (0.80, 1.23)
Vegans	9	1.14 (0.58, 2.22)	21	1.35 (0.86, 2.12)	9	1.29 (0.66, 2.52)
*p*-heterogeneity^b^		0.71		0.47		0.74
**Hip fractures**						
Meat eaters	507	Reference	536	Reference	463	Reference
Fish eaters	95	1.21 (0.96, 1.52)	97	1.19 (0.95, 1.49)	85	1.22 (0.96, 1.55)
Vegetarians	136	1.25 (1.02, 1.54)	130	1.22 (0.99, 1.50)	115	1.25 (1.01, 1.56)
Vegans	14	2.39 (1.39, 4.11)	31	2.71 (1.85, 3.95)	13	2.43 (1.38, 4.28)
*p*-heterogeneity^b^		0.003		< 0.001		0.004
**Leg fractures**						
Meat eaters	191	Reference	197	Reference	179	Reference
Fish eaters	38	0.98 (0.68, 1.42)	38	1.04 (0.73, 1.50)	34	0.97 (0.66, 1.42)
Vegetarians	61	1.00 (0.73, 1.37)	51	0.95 (0.68, 1.32)	46	0.87 (0.62, 1.24)
Vegans	7	2.50 (1.15, 5.42)	16	2.90 (1.68, 4.99)	6	2.32 (1.00, 5.34)
*p*-heterogeneity^b^		0.13		0.001		0.17
**Ankle fractures**						
Meat eaters	277	Reference	296	Reference	263	Reference
Fish eaters	49	0.84 (0.61, 1.15)	44	0.79 (0.57, 1.10)	40	0.77 (0.55, 1.09)
Vegetarians	88	0.91 (0.70, 1.19)	79	0.97 (0.74, 1.27)	72	0.92 (0.69, 1.23)
Vegans	3	0.73 (0.23, 2.31)	9	1.19 (0.60, 2.36)	3	0.85 (0.27, 2.69)
*p*-heterogeneity^b^		0.66		0.50		0.54
**Other main site fractures**^c^						
Meat eaters	249	Reference	254	Reference	229	Reference
Fish eaters	49	0.97 (0.70, 1.34)	47	0.95 (0.68, 1.31)	44	0.95 (0.68, 1.34)
Vegetarians	84	0.97 (0.74, 1.27)	75	0.93 (0.70, 1.24)	70	0.93 (0.69, 1.25)
Vegans	11	2.30 (1.23, 4.28)	18	1.82 (1.10, 3.01)	11	2.40 (1.28, 4.50)
*p*-heterogeneity^b^		0.06		0.08		0.03

^a^All analyses were stratified by sex, method of recruitment (general practice or postal), and region (7 categories), and adjusted for year of recruitment (per year from ≤ 1994 to ≥ 1999), ethnicity (white, other, unknown), Townsend deprivation index (quartiles, unknown), education level (no qualifications, basic secondary (e.g. O level), higher secondary (e.g. A level), degree, unknown), physical activity (inactive, low activity, moderately active, very active, unknown), smoking (never, former, light, heavy, unknown), alcohol consumption (< 1 g, 1–7 g, 8–15 g, 16+ g/day), dietary supplement use (no, yes, unknown), height (5 cm categories from < 155 to ≥ 185 cm, unknown), BMI (< 18.5, 18.5–19.9, 20–22.4, 22.5–24.9, 25–27.4, 27.5–29.9, 30–32.4, ≥ 32.5 kg/m^2^, unknown), and in women menopausal status (premenopausal, perimenopausal, postmenopausal, unknown), hormone replacement therapy use (never, ever, unknown), and parity (none, 1–2, ≥ 3, unknown)

^b^Represents heterogeneity in risk between diet groups based on Wald’s tests

^c^Defined as fractures of the clavicle, rib, or vertebra

In stratified analyses of total (Table 5) and hip fractures (Additional File 1: Table S6), a significantly higher risk of both total and hip fractures was only observed in vegetarians over age 50 at recruitment, although vegans had higher risks in both age groups, and a significant *p* for interaction was only observed for total fractures. For both types of fractures, the significant associations in vegans appeared stronger in women, particularly those who were postmenopausal, and participants with low physical activity and lower BMI, possibly partly due to the larger number of participants in most of these subgroups, but a higher risk of hip fracture was only observed in the fish eaters and vegetarians in the higher BMI category. Because the numbers of cases in these subgroup analyses were often very small, it is likely that we did not have sufficient power to identify possible differences.

**Table 5:** Risks of total fractures by diet group, stratified by age, sex, menopausal status, physical activity, and BMI

Stratifying variable	***N*** cases in strata, hazard ratios (95% confidence intervals)^a^				Test of interaction^b^
**Age at recruitment**		**< 50 years**		**≥ 50 years**	
Meat eaters	690	Reference	1778	Reference	
Fish eaters	204	0.86 (0.73, 1.01)	260	0.99 (0.87, 1.14)	
Vegetarians	459	1.00 (0.88, 1.13)	403	1.14 (1.01, 1.27)	*χ*^2^ = 23.07
Vegans	80	1.31 (1.03, 1.67)	67	1.50 (1.17, 1.93)	*p* < 0.001
*p*-heterogeneity^c^		0.016		0.003	
**Sex**		**Men**		**Women**	
Meat eaters	440	Reference	2028	Reference	
Fish eaters	76	0.94 (0.73, 1.21)	388	0.95 (0.85, 1.06)	
Vegetarians	202	1.06 (0.88, 1.28)	660	1.09 (0.99, 1.20)	*χ*^2^ = 6.39
Vegans	47	1.18 (0.85, 1.62)	100	1.53 (1.24, 1.88)	*p* = 0.09
*p*-heterogeneity^c^		0.64		< 0.001	
**Menopausal status**^d^		**Premenopausal**		**Postmenopausal**	
Meat eaters	167	Reference	1500	Reference	
Fish eaters	54	0.64 (0.47, 0.87)	231	1.01 (0.88, 1.17)	
Vegetarians	161	0.91 (0.73, 1.15)	325	1.13 (0.99, 1.28)	*χ*^2^ = 3.74
Vegans	27	1.41 (0.93, 2.15)	52	1.62 (1.22, 2.16)	*p* = 0.44
*p*-heterogeneity^c^		0.004		0.004	
**Physical activity**		**Inactive/low**		**Moderate/high**	
Meat eaters	1437	Reference	561	Reference	
Fish eaters	253	0.94 (0.82, 1.08)	144	0.96 (0.79, 1.16)	
Vegetarians	495	1.09 (0.98, 1.22)	257	1.03 (0.88, 1.22)	*χ*^2^ = 0.69
Vegans	79	1.43 (1.13, 1.80)	50	1.34 (0.98, 1.82)	*p* = 0.88
*p*-heterogeneity^c^		0.006		0.25	
**Body mass index**		**< 22.5 kg/m**^**2**^		**≥ 22.5 kg/m**^**2**^	
Meat eaters	762	Reference	1593	Reference	
Fish eaters	210	0.95 (0.81, 1.11)	237	0.97 (0.84, 1.11)	
Vegetarians	428	1.17 (1.02, 1.33)	396	1.04 (0.92, 1.17)	*χ*^2^ = 4.71
Vegans	95	1.66 (1.32, 2.08)	43	1.10 (0.80, 1.49)	*p* = 0.19
*p*-heterogeneity^c^		< 0.001		0.76	

^a^Results shown were for subset analyses by the stratifying variable. Analyses were stratified by sex, method of recruitment (general practice or postal), and region (7 categories), and adjusted for year of recruitment (per year from ≤ 1994 to ≥ 1999), ethnicity (white, other, unknown), Townsend deprivation index (quartiles, unknown), education level (no qualifications, basic secondary (e.g. O level), higher secondary (e.g. A level), degree, unknown), physical activity (inactive, low activity, moderately active, very active, unknown), smoking (never, former, light, heavy, unknown), alcohol consumption (< 1 g, 1–7 g, 8–15 g, 16+ g/day), dietary supplement use (no, yes, unknown), height (5 cm categories from < 155 to ≥ 185 cm, unknown), BMI (< 18.5, 18.5–19.9, 20–22.4, 22.5–24.9, 25–27.4, 27.5–29.9, 30–32.4, ≥ 32.5 kg/m^2^, unknown), and in women menopausal status (premenopausal, perimenopausal, postmenopausal, unknown), hormone replacement therapy use (never, ever, unknown), and parity (none, 1–2, ≥ 3, unknown), except the stratifying variable where appropriate

^b^Interactions by age at recruitment, sex, menopausal status, physical activity, and body mass index were investigated by including both strata in the model (e.g. both men and women) and comparing Cox models with and without the appropriate interaction term using likelihood ratio tests

^c^Represents heterogeneity in risk between diet groups based on Wald tests

^d^Premenopausal women included women who were below age 50 years at recruitment if they were perimenopausal or had unknown menopausal status; analyses in premenopausal women were censored at age 50. Postmenopausal women included women above age 50 years at recruitment if perimenopausal or had unknown perimenopausal status

## Discussion

### Summary of findings

Overall, vegans in this study had higher risks of total and some site-specific fractures (hip, leg, vertebra) than meat eaters. The strongest associations were observed for hip fractures, for which fish eaters, vegetarians, and vegans all had higher risks. These risk differences might be partially explained by the lower average BMI, and lower average intakes of calcium and protein in the non-meat eaters. However, because the differences remained, especially in vegans, after accounting for these factors, other unaccounted for factors may be important.

### Comparison with previous studies

Few previous studies have examined the associations of vegetarian diets with fracture risk. In previous EPIC-Oxford analyses of self-reported fractures with short follow-up, vegans, but not fish eaters or vegetarians, were reported to have 30% (HR 1.30; 1.02, 1.66) higher risks of total fractures, but in contrast to the current findings, the association attenuated completely when restricted to participants who reported consuming at least 525 mg/day of calcium [16]. This apparent inconsistency might be explained by several differences between the current and previous analysis; while the current analysis included close to 4000 hospital-admitted cases over more than 17 years of average follow-up on around 55,000 participants, the previous study included under 2000 self-reported fracture cases over 5 years of follow-up on around 35,000 participants. Given the difference in case ascertainment method, the current analysis is less prone to reporting error and is not susceptible to selective drop-out. It is also possible that there was insufficient power to detect a difference after stratifying by calcium intake status in the previous analysis, which also did not examine site-specific fractures.

The only other studies which reported on risks of fractures by diet groups were one small prospective study in Vietnam of 210 women (105 vegans) which found no significant difference in fracture incidence (10 cases in total) between vegans and omnivores over 2 years [17], and one prospective study in India which reported a higher crude rate of stress fractures (604 cases in total) among 2131 vegetarian than 6439 non-vegetarian army recruits [18]. Separately, previous findings from the Adventist Health Study 2, which has a large proportion of vegetarians, showed that participants who ate meat more than three times a week had lower risks of hip fractures (HR 0.60; 0.41, 0.87) than participants who ate meat less than once a week [32], while combined analyses of peri- and postmenopausal women from Adventist Health Study 1 and 2 found that participants who ate meat more than four times a week had lower risks of wrist fractures (HR 0.44; 0.23, 0.84) than participants who never ate meat [33], but these results cannot be used to infer risks in fish eaters, vegetarians, or vegans as separate diet groups.

### Interpretation of results and implications

The higher observed risks of fractures in non-meat eaters were usually stronger before BMI adjustment, which suggests that the risk differences were likely partially due to differences in BMI. Vegetarians and vegans generally have lower BMI than meat eaters [2, 8], and previous studies have reported an inverse association between BMI and some fractures, particularly hip fractures, possibly due to reasons including the cushioning against impact force during a fall, enhanced oestrogen production with increased adiposity, or stronger bones from increased weight-bearing [14, 34]. However, a positive association between BMI and fracture risk has been observed for some other sites, including ankle fractures, possibly as a result of higher torques from twisting of the ankle in people with higher BMI [14]. No significant differences in the risks of ankle fractures by diet group were observed in our study, but the point estimates were directionally consistent with a lower risk in all non-meat eaters before BMI adjustment, and the results might reflect a counterbalance between a protective effect from lower BMI but higher risk due to lower intakes of nutrients related to bone health in the non-meat eaters.

In our stratified analyses, there is limited evidence of heterogeneity in fracture risk by BMI categories. Although a statistically significant higher risk of total and hip fractures was only observed in vegans in the lower BMI category (< 22.5 kg/m^2^), our interpretation is limited by the small numbers of cases in each stratum in these analyses, especially because of the strong correlation between diet group and BMI, which results in very few vegans in the higher BMI category, and vice versa comparatively small numbers of meat eaters with a low BMI. In addition to BMI, previous studies have reported that muscle strength is an important risk factor which is protective against fall risk and subsequently fractures in older adults [35]. A previous study in the UK found lower lean mass and grip strength in vegetarians and vegans compared to meat eaters [2]; therefore, the possible influences of muscle strength and fall risk in addition to bone health on fracture risk in vegetarian and vegan populations should be further investigated. Fractures at some sites, especially at the hip, may also be more related to osteoporosis than fractures at some other sites, which might be more likely to be the result of violent impacts in accidents [36, 37]. We were unable to differentiate fragility and traumatic fractures in this study, since data were not available on the causes of the fractures.

In this study and previous studies, vegans had substantially lower intakes of calcium than other diet groups since they do not consume dairy, a major source of dietary calcium [4, 5], while both vegetarians and vegans had lower protein intakes on average [6, 7]. In the human body, 99% of calcium is present in bones and teeth in the form of hydroxyapatite, which in cases of calcium deficiency gets resorbed to maintain the metabolic calcium balance, and thus, osteoporosis could occur if the calcium was not restored [38–40]. A recent meta-analysis reported that increasing calcium intake from either dietary sources or supplements resulted in small increases in BMD [9], but the evidence on fracture risk has been less consistent. Previous analyses in EPIC-Oxford found a higher risk of self-reported fractures in women, but not men, with calcium intakes below 525 mg/day compared with over 1200 mg/day [41]. A recent meta-analysis of both randomised trials and prospective studies concluded that there was no evidence of an association between calcium intake from diet and fracture risk, but a possible weak protective association between calcium supplement use and some fractures [10]. More recently however, a separate meta-analysis showed a protective effect against fractures of combined vitamin D and calcium supplements, but not vitamin D supplements alone [11].

For protein, some older studies suggested that excessive protein intake would lead to an increased metabolic acid load, subsequently buffered by bone resorption and calciuria, and thus poorer bone health [12, 42]. However, more recent experimental evidence has shown that high protein intake also increases intestinal calcium absorption [43], and stimulates the production of insulin-like growth factor (IGF)-I [44], which in turn is associated with better bone health [45, 46]. Two meta-analyses, which included different studies, both reported a possible protective effect of higher protein intake on lumbar spine BMD [13, 47]; several epidemiological studies have reported inverse associations between protein intake and fracture risks [48–50], though a recent meta-analysis found no significant association between protein intake and osteoporotic fractures [51].

The higher risks of fractures especially in the vegans remained significant after adjustment for dietary calcium and protein, which suggests that these factors may at most only partly explain the differences in fracture risks by diet group, and other factors may also contribute. However, estimation of intakes of these nutrients by questionnaires has substantial error, and we were only able to account for differences in dietary calcium but not differences in calcium supplement use, since data on the latter were not available. A detailed analysis of the associations of specific foods, such as meat or dairy, with fracture risk is beyond the scope of the current study, but should be explored in further studies. Future research should also focus on possible effects of other nutrients or biological markers on fracture risks, for example circulating vitamin D, vitamin B_12_, or IGF-I, which may vary by degree of animal-sourced food intake [52–54]. The value of incorporating habitual dietary habits in addition to established parameters for predicting fracture risks in clinical settings should also be further explored.

### Strengths and limitations

The strengths of this study were that it included a large number of non-meat eaters with a long follow-up, and studied both total and site-specific fractures, after accounting for a range of confounders. We updated diet group and relevant confounders where possible, to account for changes over the period of follow-up. There was little evidence of reverse causality, as results were similar after excluding the first 5 years of follow-up. The outcome data were ascertained based on hospital records, which reduced misreporting and selective loss to follow-up, although a possible limitation of this approach was that less serious fractures that did not require hospitalisation would not have been captured.

Of other limitations, while we excluded known cases of fractures before baseline based on hospital records, this may not be a complete exclusion, since no questions on previous diagnosis of fractures (prior to the earliest available hospital data) or osteoporosis were asked at baseline, and no data on the use of anti-osteoporosis medication were available. Repeat measures of diet were not available in all participants, and the exact date of dietary change during follow-up was also not recorded, but considering the good agreement of diet group in participants who did provide a repeat measure, and the fact that a dietary change may only influence fracture risk after a period of time, we do not expect substantial misclassification. As with all observational studies, residual confounding from both dietary and non-dietary factors may be present; for example, the role of calcium might have been underestimated due to measurement error. As the study predominantly includes white European participants, generalisability to other populations or ethnicities may be limited, which could be important considering previously observed differences in BMD [2, 55] and fracture risks [56] by ethnicity. We also observed only a small number of cases in many subgroup analyses, and thus, it is likely we had insufficient power to reliably assess whether there might be any heterogeneity by these subgroups including age, sex, menopausal status, or BMI; additional data are therefore needed to confirm or refute possible differences. In particular, because the EPIC-Oxford cohort consists predominantly of women (77%), further work should be conducted in cohorts with a larger proportion of men to explore heterogeneity by sex and to derive reliable sex-specific estimates.

## Conclusions

Overall, we found that compared with meat eaters, vegans had higher risks of total, hip, leg, and vertebral fractures, while fish eaters and vegetarians had higher risk of hip fractures. These risk differences were likely partly due to their lower BMI, and possibly to lower intakes of calcium and protein. More studies are needed especially from non-European and contemporary populations to examine the generalisability of our findings and to explore possible heterogeneity by factors including age, sex, menopausal status, and BMI. Future work might benefit from examining possible biological pathways by investigating serum levels of vitamin D, vitamin B_12_, or IGF-1, or in assessing the possible roles of other nutrients that are abundant in animal-sourced foods.

## Supplementary information

**Additional file 1: **Supplementary results. **Fig. S1.** Participant flow chart. **Table S1.** ICD codes for incident fractures. **Table S2.** Baseline characteristics by diet group and sex. **Table S3.** Food and nutrient intake by diet group and sex. **Table S4.** Risks of subtypes of main site fractures. Table S5-Sensitivity analyses. **Table S6.** Risks of hip fractures by age, sex, menopausal status, physical activity and BMI.

## Data Availability

The data access policy for the EPIC-Oxford study is available via the study website (www.epic-oxford.org/data-access-sharing-and-collaboration/).

## Abbreviations

BMD: Bone mineral density
BMI: Body mass index
EPIC: European Prospective Investigation into Cancer and Nutrition
ICD: International Classification of Diseases
IGF-1: Insulin-like growth factor-1
