# Supplementary material for: Vegetarian and vegan diets and risks of total and site-specific fractures: results from the prospective EPIC-Oxford study
Source: BMC Med. 2020 Nov 23;18:353. doi: 10.1186/s12916-020-01815-3 (PMC7682057; doi:10.1186/s12916-020-01815-3)
Supplement: Supplementary file 1 — Additional file 1: Supplementary results. Fig. S1. Participant flow chart. Table S1. ICD codes for incident fractures. Table S2. Baseline characteristics by diet group and sex. Table S3. Food and nutrient intake by diet group and sex. Table S4. Risks of subtypes of main site fractures. Table S5-Sensitivity analyses. Table S6. Risks of hip fractures by age, sex, menopausal status, physical activity and BMI. [file 12916_2020_1815_MOESM1_ESM.docx]

**Additional file 1**

[**Supplementary methods** 2](#_Toc53059020)

[**Supplementary results** 4](#_Toc53059021)

[**Fig. S1**: Participant flow chart of the study. 5](#_Toc53059022)

[**Table S1**: ICD codes for identifying incident fractures. 6](#_Toc53059023)

[**Table S2**: Baseline characteristics of EPIC-Oxford participants by diet group and sex. 7](#_Toc53059024)

[**Table S3**: Intake of selected food and nutrients of EPIC-Oxford participants by diet group and sex. 8](#_Toc53059025)

[**Table S4**: Risks for subtypes of main site fractures by diet groups in EPIC Oxford. 9](#_Toc53059026)

[**Table S5**: Sensitivity analyses of risks of total and site-specific fractures by diet group in EPIC-Oxford. 10](#_Toc53059027)

[**Table S6**: Risks of hip fractures by diet group, stratified by age, sex, menopausal status, physical activity and BMI. 12](#_Toc53059028)

**Supplementary methods**

*EPIC-Oxford recruitment*

Details of the EPIC-Oxford recruitment process have been described previously [4]. In brief, two recruitment methods were used, including a general practice (GP) recruitment and a postal recruitment. The GP recruitment method recruited 7421 men and women aged 35 to 59 years who were registered with participating GPs, all of whom completed a full questionnaire on their diet, lifestyle, health characteristics and medical history. The postal recruitment preferentially targeted vegetarians, vegans, and other people interested in diet and health, and recruited 57 990 participants aged ≥20 years. A full questionnaire was mailed to all members of the Vegetarian Society and all surviving participants of the Oxford Vegetarian Study [19], and respondents were invited to provide names and addresses of relatives and friends who were also interested in receiving a questionnaire. A short questionnaire was also distributed to all members of the Vegan Society, enclosed in vegetarian and health food magazines, and displayed in health-food shops, and a full questionnaire was subsequently mailed to all those who returned the short questionnaire. Subsequently, a follow-up questionnaire which asked similar questions on diet and lifestyle was sent to participants in 2010. Additional details and numbers at each stage of the recruitment process is shown as a flow chart in Figure S1.

*Dietary questionnaires and classification of diet group*

Both the full baseline questionnaire and follow-up questionnaire in 2010 collected responses to the same four questions about consumption of meat, fish, dairy products, and eggs, in the form of “Do you eat any meat (including bacon, ham, poultry, game, meat pies, sausages)?” or similar for the other three food groups. These four questions were used to classify participants into one of four diet groups at baseline and where available also at follow-up: meat eaters (participants who reported eating meat, including red meat, processed meat, and poultry, regardless of whether they ate fish, dairy, or eggs), fish eaters (participants who did not eat meat but did eat fish); vegetarians (participants who did not eat meat or fish, but did eat one or both of dairy products and eggs); and vegans (participants who did not eat meat, fish, dairy products and eggs).

Information from both the baseline questionnaire and follow-up questionnaire was used for diet group classification. For participants who reported a change in diet group at follow-up, risk from baseline to the return of the follow-up questionnaire was assessed using the baseline diet group classification, while risk in the period after the return of the follow-up questionnaire was assessed using the new diet group classification at follow-up. For participants who did not complete the follow-up questionnaire, the baseline diet group classification was used to assess risk for the whole follow-up period. The agreement of diet group among 30,391 participants with measurements at both baseline and follow-up was good. Of 16,175 meat eaters at baseline, 15,571 (96%) remained meat eaters at follow-up, 449 (2.8%) became fish eaters, 145 (0.9%) became vegetarians, 10 (0.06%) became vegans. Of 4,687 fish eaters at baseline, 2,694 (57%) remained fish eaters at follow-up, 1,584 (34%) became meat eaters, 393 (8.4%) became vegetarians, 16 (0.3%) became vegans. Of 8,649 vegetarians at baseline, 5,929 (69%) remained vegetarians at follow-up, 1,107 (13%) became meat eaters, 1,332 (15%) became fish eaters, 281 (3.3%) became vegans. Of 880 vegans at baseline, 489 (56%) remained vegans at follow-up, 86 (9.8%) became meat eaters, 75 (8.5%) became fish eaters, 230 (26%) became vegetarians.

Additionally, for participants who reported ‘no’ to the questions “Do you eat any meat” or similar for the other three food groups, an additional question was asked “If no, how old were you when you last ate meat?”, or similar for the other food groups. The majority of participants had been in their respective diet groups for substantial periods before recruitment. For example, 88% of fish eaters, vegetarians and vegans had not eaten meat for at least 5 years before recruitment, and 85% of vegetarians and vegans had not eaten fish for at least 5 years before recruitment. In vegans, 61% had not eaten eggs or dairy for at least 5 years, and 82% had not eaten eggs or dairy for at least 3 years.

Furthermore, the baseline questionnaire included a validated 130-item semi-quantitative food-frequency section which asked about dietary intake over the past year [20-22]. The follow-up questionnaire sent in 2010 included a 112-item food frequency section which also asked about dietary intake over the past year. The follow-up questionnaire included additional items on specific vegetarian or vegan foods, such as vegan margarine or vegan burgers, and combined some food items which were separated on the baseline questionnaire e.g. peas and beans were combined as one item. For calculation of food and nutrient intakes, the frequency of consumption of each food or beverage was multiplied by a standard portion size (mostly based on UK Ministry of Agriculture, Fisheries and Food data) [23] and the nutrient content of each food or beverage (based on McCance and Widdowson’s food composition tables) [24].

*Collection of and categorisation of covariates*

In the full recruitment questionnaire, apart from diet, participants were also asked questions on socio-demographic characteristics and lifestyle and medical history, including ethnicity, education level, smoking, physical activity, use of dietary supplements, and for women, use of hormone replacement therapy, number of children, and questions relating to their menstrual status. Alcohol consumption was determined from responses to 5 items on the food frequency questionnaire. Socio-economic status was categorised using the Townsend deprivation index [25], based on the participants’ postcodes. For physical activity, participants were categorised using a validated four-level physical activity index based on responses to questions asked about occupation and time spent participating in activities including walking, cycling, and other physical exercise [26]. Questions relating to smoking and alcohol consumption were also asked on the follow-up questionnaire in 2010. BMI was calculated from participants’ self-reported height and weight at recruitment and weight at follow-up; the self-reported measures were previously found to be sufficiently accurate compared to measured height and weight in a validation study of about 4800 participants [27].

For the analyses, the covariates were categorised as follows. All analyses were stratified by sex, method of recruitment (general practice or postal), and region of residence (7 regions across the UK), and adjusted for year of recruitment (per year from ≤1994 to ≥1999), ethnicity (white, other, unknown), Townsend deprivation index (quartiles, unknown)[8], education level (no qualifications, basic secondary (e.g. O level), higher secondary (e.g. A level), degree, unknown), physical activity (inactive, low activity, moderately active, very active, unknown)[9], smoking (never, former, light, heavy, unknown), alcohol consumption (<1, 1-7, 8-15, 16+ g/day), dietary supplement use (no, yes, unknown), height (5 cm categories from <155 to ≥185 cm, unknown) and in women menopausal status (premenopausal, perimenopausal, postmenopausal, unknown), hormone replacement therapy use (never, ever, unknown), and parity (none, 1-2, ≥3, unknown). BMI categories were <18.5, 18.5-19.9, 20-22.4, 22.5-24.9, 25-27.4, 27.5-29.9, 30-32.4, ≥32.5 kg/m^2^, unknown. Dietary calcium intake categories were <525, 525-699 ,700-899, 900-1199, ≥1200 mg/day, and dietary protein categories were <13, 13-14.4, 14.5-15.9, 16-18.0, ≥18.1 percent of total energy, representing approximate fifths of intake at baseline. The percentages of missing values in the covariates were 14% for physical activity, 13% for the Townsend deprivation index, 7% for education level, 3% for BMI, and 2% or less for each of the other covariates.

*Exclusion criteria*

Participants were excluded if they resided outside England, Wales, or Scotland (n=947), had no Hospital Episode Statistics data or National Health Service number (n=20), completed the short questionnaire only (n=7619), were younger than 20 years or older than 90 years at recruitment (n=59), had no follow-up (i.e. were censored at or before the date of recruitment, for example if they were living abroad, n=364), could not be traced by the National Health Service (n=14), did not answer the relevant questions to be classified into a diet group (n=132), had unreliable nutrient data (i.e. ≥20% of food frequencies missing, or daily energy intakes <500 kcal or >3500 kcal for women or <800 kcal or >4000 kcal for men, n=1219), or had a date of diagnosis which preceded or equalled the date of recruitment (varying numbers for each fracture site, details in Figure S1).

**Supplementary results**

*Baseline characteristics*

Baseline characteristics in the overall cohort are presented in Table 1. Overall, compared with meat eaters, non-meat eaters were younger and less likely to be in the top socio-economic quartile, but reported higher education level, lower current smoking levels, and higher physical activity levels. The vegetarians and vegans reported slightly lower average alcohol consumption, while the fish eaters reported higher and the vegans reported lower dietary supplement use than the meat eaters.

In terms of health characteristics, compared with meat eaters, the other diet groups had lower BMI on average, and among women were more likely to be premenopausal, less likely to report hormone replacement therapy use and less likely to have had any children. The distribution of baseline characteristics by diet group were mostly similar when tabulating separately by men and women (Additional File 1: Table S2), with the exception that in men the meat eaters had the highest average alcohol consumption while in women the meat eaters had the lowest average alcohol consumption, but the differences between diet group was small.

For diet (Table 1, Additional File 1: Tables S2 and S3), total energy was lower in the other diet groups compared with meat eaters, and they also reported lower total protein but higher plant protein as a percentage of total energy. Vegans showed much lower dietary calcium intake than all other diet groups. Both vegan men and women had high intake of fruit and vegetables, legumes and soya, nuts and nut butter, and a high % energy intake from carbohydrates and higher dietary fibre intake than the meat eaters, but they had lower total fat intake.

*Secondary analyses*

In analyses limited to participants who met either or both dietary guidelines for calcium and protein intakes, all estimates were similar, and in cases where the associations became attenuated (e.g. risks of leg fractures in vegans), the confidence intervals were much wider likely due to the smaller number of cases (Table 4).

In sensitivity analyses additionally adjusting for total energy, excluding the first five years of follow-up, excluding prior diseases, excluding people receiving long-term treatment for any illness, and including multiple imputation for missing covariates, results were similar but confidence intervals were sometimes wider, suggesting a loss of statistical power due to the smaller sample size in some of the analyses (Additional File 1: Table S5).

65,411 participants recruited between 1993 and 2001, and provided informed consent:

- 7,421 from GP recruitment
- 57,990 from postal recruitment

Exclusion criteria:

- Not resident in England, Wales, or Scotland (n=947)
- No Hospital Episode Statistics data or National Health Service number (n=20)
- Completed the short questionnaire only (n=7,619)
- Were younger than 20 years at recruitment (n=1)
- Were older than 90 years at recruitment (n=58)
- Had no follow up (n=364)
- Could not be traced by the National Health Service (n=14)
- Of unknown diet group (n=132)
- Had unreliable nutrient data (n=1,219)
- Had a date of diagnosis which preceded or equalled the date of recruitment

- Total fractures (n=139)

- Arm fractures (n=23)

- Wrist fractures (n=24)

- Hip fractures (n=11)

- Leg fractures (n=18)

- Ankle fractures (n=16)

- Other main sites (n=21)

54,898 participants for total fractures, including:

- 7,176 from GP recruitment
- 7022 meat eaters
- 81 fish eaters
- 73 vegetarians
- 0 vegans
- 47,722 from postal recruitment
- 22,358 meat eaters
- 7,956 fish eaters
- 15,426 vegetarians
- 1,982 vegans

Of which 30,391 participants returned a follow-up questionnaire in 2010 to 2013

Total number/number with repeated measures for the site specific fractures:
- Arm fractures (55,014/31,476)
- Wrist fractures (55,013/31,339)
- Hip fractures (55,026/31,487)
- Leg fractures (55,019/31,525)
- Ankle fractures (55,021/31,458)
- Other main site fractures (55,016/31,539)

**Fig. S1**: Participant flow chart of the study.

**Table S1**: ICD codes for identifying incident fractures.

| Outcome | ICD code |
| --- | --- |
| Total fracture | ICD-9: 800-829 / ICD-10: S02, S12, S22, S32, S42, S52, S62, S72, S82, S92, T02, T08, T10, T12 |
| Arm fracture | ICD-9: 812, 813.0-813.3, 813.8-813.9 / ICD-10: S42.2-S42.4, S52.0-S52.4, S52.7 |
| Wrist fracture | ICD-9: 813.4-813.5, 814 / ICD-10: S52.5-52.6, S62.0-62.1, S62.8 |
| Hip fracture | ICD-9: 820 / ICD-10: S72.0-S72.2 |
| Leg fracture | ICD-9: 821, 822, 823 / ICD-10: S72.3-S72.4, S82.0, S82.1-82.2, 82.4 |
| Ankle fracture | ICD-9: 824 / ICD-10: S82.3, 82.5-82.6, 82.8 |
| Other main site fractures i.e. clavicle, rib, or vertebra | Clavicle; ICD-9: 810 / ICD-10: S420, rib; ICD-9: 807.0, 807.1, 807.4 / ICD-10: S22.3-22.5, vertebra; ICD-9: 805-806 / ICD-10: S12.0-12.2, 12.7, 22.0-22.1, S32.0-32.2 |

**Table S2**: Baseline characteristics of EPIC-Oxford participants by diet group and sex.

| **Characteristics** | **Diet group** | | | |
| --- | --- | --- | --- | --- |
| **Mean (SD) or n (%)** | **Meat eaters** | **Fish eaters** | **Vegetarians** | **Vegans** |
| **Men** | **n=6789** | **n=1423** | **n=3588** | **n=716** |
| **Socio-demographic** |  |  |  |  |
| Age, years (SD) | 52.4 (13.6) | 45.5 (13.5) | 43.0 (14.0) | 40.5 (13.4) |
| Top socio-economic quartile (%)^a^ | 1787 (29.6) | 234 (19.1) | 661 (21.0) | 117 (18.4) |
| Higher education (%) | 2552 (41.3) | 760 (55.6) | 1740 (51.1) | 299 (44.4) |
| White ethnicity (%) | 6570 (98.6) | 1354 (96.4) | 3357 (96.1) | 658 (96.3) |
| **Lifestyle** |  |  |  |  |
| Current smokers, (%) | 993 (14.7) | 187 (13.2) | 408 (11.4) | 73 (10.2) |
| Alcohol consumption, g/day (SD) | 16.2 (18.1) | 16.1 (17.7) | 14.3 (17.9) | 11.5 (17.4) |
| Moderate or high physical  activity, (%) | 2192 (35.5) | 598 (46.3) | 1461 (44.5) | 344 (52.3) |
| Dietary supplement use, (%)^b^ | 2869 (43.0) | 689 (49.4) | 1597 (45.0) | 306 (43.8) |
| **Health characteristics and medical history** |  |  |  |  |
| Body mass index, kg/m^2^ (SD) | 24.9 (3.3) | 23.5 (3.0) | 23.5 (3.2) | 22.5 (3.0) |
| <20 kg/m^2^ (%) | 259 (3.9) | 105 (7.6) | 336 (9.8) | 118 (17.1) |
| ≥25 kg/m^2^ (%) | 2834 (43.2) | 343 (24.9) | 905 (26.3) | 112 (16.2) |
| **Dietary information** |  |  |  |  |
| Energy, kJ/day (SD) | 9,177 (2,458) | 8,912 (2,424) | 8,784 (2,384) | 8,020 (2,431) |
| Dietary calcium, mg/day (SD) | 1,058 (331) | 1,088 (367) | 1,089 (409) | 611 (239) |
| Protein, % energy (SD) | 16.0 (2.8) | 13.9 (2.2) | 13.0 (1.9) | 12.9 (2.2) |
|  |  |  |  |  |
| **Women** | **n=22591** | **n=6614** | **n=11911** | **n=1266** |
| **Socio-demographic** |  |  |  |  |
| Age, years (SD) | 49.4 (12.9) | 42.1 (13.2) | 39.1 (13.2) | 38.1 (13.7) |
| Top socio-economic quartile (%)^a^ | 5160 (26.7) | 1300 (22.5) | 2291 (22.0) | 191 (17.2) |
| Higher education (%) | 5646 (27.6) | 2697 (43.1) | 4549 (40.2) | 498 (41.9) |
| White ethnicity (%) | 21764 (98.6) | 6363 (98.0) | 11407 (97.7) | 1190 (97.3) |
| **Lifestyle** |  |  |  |  |
| Current smokers, (%) | 2630 (11.7) | 618 (9.4) | 1146 (9.7) | 139 (11.0) |
| Alcohol consumption, g/day (SD) | 7.7 (9.7) | 8.5 (10.2) | 7.7 (9.9) | 6.5 (10.2) |
| Moderate or high physical  activity, (%) | 5324 (29.0) | 2197 (37.9) | 3939 (37.3) | 463 (41.2) |
| Dietary supplement use, (%)^b^ | 13072 (59.2) | 4357 (67.5) | 7063 (60.5) | 702 (56.4) |
| **Health characteristics and medical history** |  |  |  |  |
| Body mass index, kg/m^2^ (SD) | 24.3 (4.2) | 22.8 (3.5) | 22.7 (3.5) | 21.9 (3.0) |
| <20 kg/m^2^ (%) | 2106 (9.6) | 1073 (16.8) | 2172 (19.0) | 338 (27.5) |
| ≥25 kg/m^2^ (%) | 7573 (34.6) | 1259 (19.7) | 2177 (19.0) | 156 (12.7) |
| **Dietary information** |  |  |  |  |
| Energy, kJ/day (SD) | 8,018 (2,106) | 7,730 (2,101) | 7,589 (2,081) | 6,959 (2,155) |
| Dietary calcium, mg/day (SD) | 989 (307) | 1,021 (343) | 1,012 (355) | 580 (235) |
| Protein, % energy (SD) | 17.3 (3.0) | 14.8 (2.3) | 13.8 (2.1) | 13.5 (2.3) |

Estimates shown are mean (SD) or numbers (%), as stated in left column. Percentages were estimated excluding participants with missing responses.
^a^ Based on Townsend deprivation index.
^b^ Defined as regularly taking any vitamins, minerals, fish oils, fibre or other food supplements during the last 12 months.

**Table S3**: Intake of selected food and nutrients of EPIC-Oxford participants by diet group and sex.

| **Foods or nutrients** | **Diet group** | | | |
| --- | --- | --- | --- | --- |
| **Mean (SD)** | **Meat eaters** | **Fish eaters** | **Vegetarians** | **Vegans** |
| **Men** | **n=6789** | **n=1423** | **n=3588** | **n=716** |
| Total meat and meat products (g/day) | 85.7 (53.9) | 1.8 (11.1) | 0.4 (5.0) | 0.4 (6.6) |
| Red and processed meat (g/day) | 61.3 (45.2) | 1.2 (8.3) | 0.3 (3.5) | 0.3 (5.0) |
| Poultry (g/day) | 24.4 (22.1) | 1.6 (3.8) | 0.1 (1.9) | 0.1 (2.1) |
| Total fish and fish products (g/day) | 41.3 (30.0) | 41.1 (35.7) | 0.4 (3.6) | 0.3 (4.1) |
| Diary milk (mL/day) | 355.4 (198.7) | 286.8 (202.0) | 278.1 (230.8) | 4.8 (35.2) |
| Soya milk (mL/day) | 4.2 (36.0) | 22.3 (83.8) | 42.7 (113.7) | 236.2 (198.4) |
| Dairy cheese (g/day) | 18.7 (16.8) | 25.8 (21.4) | 29.0 (25.1) | 0.9 (3.7) |
| Total fresh fruit (g/day) | 220.6 (185.1) | 251.2 (201.6) | 241.6 (205.7) | 336.2 (300.5) |
| Total vegetables (g/day) | 224.9 (120.4) | 266.4 (137.9) | 267.6 (141.8) | 319.9 (173.3) |
| Legumes and soya (g/day) | 30.3 (30.9) | 62.2 (52.7) | 74.8 (63.0) | 110.0 (82.2) |
| Nuts and nut butter (g/day) | 5.8 (11.7) | 10.6 (16.0) | 12.3 (17.7) | 24.5 (29.4) |
| Soft drinks (g/day) | 31.1 (81.8) | 21.6 (60.0) | 26.3 (63.9) | 24.1 (75.6) |
| Low calorie or diet soft drinks (g/day) | 39.9 (112.3) | 39.9 (121.7) | 44.1 (121.2) | 30.6 (105.1) |
| Carbohydrates (% energy) | 46.9 (6.5) | 49.8 (6.6) | 51.2 (6.8) | 54.6 (7.7) |
| Total fat (% energy) | 31.9 (5.8) | 31.1 (6.1) | 31.1 (6.2) | 28.5 (7.1) |
| Dietary fibre (g/day)^a^ | 18.5 (6.8) | 21.7 (7.6) | 22.2 (7.6) | 26.8 (9.0) |
|  |  |  |  |  |
| **Women** | **n=22591** | **n=6614** | **n=11911** | **n=1266** |
| Total meat and meat products (g/day) | 75.6 (47.4) | 2.0 (10.6) | 0.4 (5.9) | 0.2 (2.7) |
| Red and processed meat (g/day) | 48.8 (37.4) | 1.0 (6.4) | 0.3 (3.8) | 0.1 (1.3) |
| Poultry (g/day) | 26.8 (22.2) | 1.9 (5.4) | 0.2 (2.5) | 0.1 (1.7) |
| Total fish and fish products (g/day) | 42.6 (29.1) | 38.2 (33.0) | 0.7 (5.4) | 0.6 (5.0) |
| Diary milk (mL/day) | 317.9 (179.8) | 271.3 (186.9) | 255.3 (192.9) | 8.6 (50.6) |
| Soya milk (mL/day) | 5.8 (42.6) | 20.9 (78.8) | 29.7 (92.7) | 220.9 (182.1) |
| Dairy cheese (g/day) | 21.2 (19.1) | 27.7 (24.6) | 30.3 (25.1) | 1.9 (8.3) |
| Total fresh fruit (g/day) | 276.1 (209.2) | 302.6 (235.6) | 287.4 (225.1) | 361.2 (373.7) |
| Total vegetables (g/day) | 259.3 (134.9) | 294.1 (152.7) | 295.1 (163.5) | 361.5 (200.3) |
| Legumes and soya (g/day) | 28.1 (29.7) | 57.4 (43.8) | 69.2 (52.8) | 102.4 (66.2) |
| Nuts and nut butter (g/day) | 4.2 (8.1) | 7.5 (11.0) | 8.6 (12.7) | 17.1 (23.5) |
| Soft drinks (g/day) | 20.6 (67.9) | 13.7 (46.7) | 17.8 (58.7) | 13.7 (47.0) |
| Low calorie or diet soft drinks (g/day) | 59.8 (144.6) | 50.6 (126.5) | 68.1 (155.0) | 49.1 (135.9) |
| Carbohydrates (% energy) | 48.3 (6.1) | 51.3 (6.4) | 52.9 (6.5) | 56.1 (7.8) |
| Total fat (% energy) | 31.5 (5.9) | 30.7 (6.4) | 30.3 (6.6) | 27.8 (7.4) |
| Dietary fibre (g/day)^a^ | 18.8 (6.7) | 21.2 (7.5) | 21.4 (7.8) | 25.7 (9.4) |

^a^ Estimated as non-starch polysaccharides.

**Table S4**: Risks for subtypes of main site fractures by diet groups in EPIC Oxford.

| **Fracture site/diet group** | **N Cases** | **Age at event in cases, mean years (SD)** | **Hazard ratios (95% confidence intervals)**^a^ |
| --- | --- | --- | --- |
| **Clavicle fracture** |  |  |  |
| Meat eaters | 45 | 61.9 (16.2) | Reference |
| Fish eaters | 19 | 61.5 (15.1) | 1.56 (0.89,2.74) |
| Vegetarians | 28 | 54.8 (17.3) | 1.22 (0.73,2.04) |
| Vegans | 7 | 51.9 (9.6) | 1.93 (0.82,4.54) |
| p-heterogeneity^c^ |  |  | 0.29 |
|  |  |  |  |
| **Rib fracture** |  |  |  |
| Meat eaters | 116 | 61.9 (16.2) | Reference |
| Fish eaters | 18 | 61.5 (15.1) | 0.83 (0.49,1.40) |
| Vegetarians | 27 | 54.8 (17.3) | 0.75 (0.47,1.18) |
| Vegans | 3 | 51.9 (9.6) | 0.57 (0.18,1.85) |
| p-heterogeneity^c^ |  |  | 0.53 |
|  |  |  |  |
| **Vertebral fracture** |  |  |  |
| Meat eaters | 126 | 69.9 (13.3) | Reference |
| Fish eaters | 20 | 62.1 (16.2) | 0.84 (0.51,1.37) |
| Vegetarians | 45 | 61.7 (16.7) | 1.09 (0.75,1.60) |
| Vegans | 13 | 59.0 (18.2) | 2.42 (1.31,4.48) |
| p-heterogeneity^b^ |  |  | 0.023 |

^a^ All analyses were stratified by sex, method of recruitment (general practice or postal), and region (7 categories), and adjusted for year of recruitment (per year from ≤1994 to ≥1999), ethnicity (white, other, unknown), Townsend deprivation index (quartiles, unknown), education level (no qualifications, basic secondary (e.g. O level), higher secondary (e.g. A level), degree, unknown), physical activity (inactive, low activity, moderately active, very active, unknown), smoking (never, former, light, heavy, unknown), alcohol consumption (<1g, 1-7g, 8-15g, 16+ g/day), dietary supplement use (no, yes, unknown), height (5cm categories from <155 to ≥185cm, unknown), BMI (<18.5, 18.5-19.9, 20-22.4, 22.5-24.9, 25-27.4, 27.5-29.9, 30-32.4, ≥32.5 kg/m^2^, unknown), and in women menopausal status (premenopausal, perimenopausal, postmenopausal, unknown), hormone replacement therapy use (never, ever, unknown), and parity (none, 1-2, ≥3, unknown).
^b^ Represents heterogeneity in risk between diet groups based on Wald tests.

**Table S5**: Sensitivity analyses of risks of total and site-specific fractures by diet group in EPIC-Oxford.

| **Fracture site/diet group** | **Additionally adjusting for total energy intake** | | **Excluding first five years of**  **follow-up** | | **Excluding prior diseases**^a^ | | **Excluding people receiving long-term treatment for any illness** | | **With multiple imputation for missing covariates** | |
| --- | --- | --- | --- | --- | --- | --- | --- | --- | --- | --- |
|  | **N Cases** | **HR (95% CI)**^b^ | **N Cases** | **HR (95% CI)**^b^ | **N Cases** | **HR (95% CI)**^b^ | **N Cases** | **HR (95% CI)**^b^ | **N Cases** | **HR (95% CI)**^b^ |
| **Total fractures** |  |  |  |  |  |  |  |  |  |  |
| Meat eaters | 2,468 | Reference | 2,053 | Reference | 1,784 | Reference | 1,285 | Reference | 2,468 | Reference |
| Fish eaters | 464 | 0.95 (0.85,1.05) | 392 | 0.99 (0.88,1.10) | 393 | 0.97 (0.87,1.09) | 299 | 0.93 (0.82,1.06) | 464 | 0.95 (0.86, 1.06) |
| Vegetarians | 862 | 1.09 (1.00,1.19) | 716 | 1.14 (1.04,1.26) | 749 | 1.12 (1.02,1.23) | 601 | 1.09 (0.98,1.21) | 862 | 1.10 (1.01, 1.19) |
| Vegans | 147 | 1.42 (1.19,1.69) | 116 | 1.47 (1.21,1.78) | 127 | 1.42 (1.18,1.71) | 121 | 1.58 (1.30,1.92) | 147 | 1.44 (1.21, 1.72) |
| p-heterogeneity^c^ |  | <0.001 |  | <0.001 |  | <0.001 |  | <0.001 |  | <0.001 |
|  |  |  |  |  |  |  |  |  |  |  |
| **Arm fractures** |  |  |  |  |  |  |  |  |  |  |
| Meat eaters | 352 | Reference | 297 | Reference | 249 | Reference | 181 | Reference | 352 | Reference |
| Fish eaters | 62 | 0.93 (0.70,1.23) | 50 | 0.90 (0.66,1.23) | 50 | 0.91 (0.66,1.25) | 39 | 0.89 (0.62,1.27) | 62 | 0.93 (0.70, 1.23) |
| Vegetarians | 130 | 1.26 (1.01,1.57) | 117 | 1.37 (1.08,1.74) | 109 | 1.24 (0.97,1.58) | 85 | 1.16 (0.88,1.54) | 130 | 1.25 (1.00, 1.56) |
| Vegans | 22 | 1.58 (1.00,2.48) | 16 | 1.41 (0.84,2.38) | 18 | 1.44 (0.87,2.38) | 18 | 1.63 (0.98,2.73) | 22 | 1.57 (1.00, 2.46) |
| p-heterogeneity^c^ |  | 0.042 |  | 0.021 |  | 0.14 |  | 0.15 |  | 0.05 |
|  |  |  |  |  |  |  |  |  |  |  |
| **Wrist fractures** |  |  |  |  |  |  |  |  |  |  |
| Meat eaters | 565 | Reference | 476 | Reference | 415 | Reference | 312 | Reference | 565 | Reference |
| Fish eaters | 110 | 0.90 (0.73,1.11) | 95 | 0.95 (0.75,1.19) | 97 | 0.95 (0.76,1.20) | 74 | 0.88 (0.68,1.15) | 110 | 0.90 (0.73, 1.12) |
| Vegetarians | 185 | 0.99 (0.83,1.19) | 162 | 1.08 (0.89,1.31) | 155 | 0.99 (0.81,1.20) | 126 | 0.96 (0.76,1.19) | 185 | 1.00 (0.84, 1.20) |
| Vegans | 29 | 1.22 (0.83,1.80) | 23 | 1.23 (0.80,1.90) | 24 | 1.18 (0.77,1.81) | 25 | 1.36 (0.89,2.08) | 29 | 1.25 (0.85, 1.84) |
| p-heterogeneity^c^ |  | 0.50 |  | 0.59 |  | 0.83 |  | 0.32 |  | 0.47 |
|  |  |  |  |  |  |  |  |  |  |  |
| **Hip fractures** |  |  |  |  |  |  |  |  |  |  |
| Meat eaters | 610 | Reference | 552 | Reference | 410 | Reference | 258 | Reference | 610 | Reference |
| Fish eaters | 122 | 1.25 (1.02,1.53) | 106 | 1.22 (0.99,1.52) | 101 | 1.36 (1.09,1.71) | 76 | 1.47 (1.13,1.92) | 122 | 1.27 (1.04, 1.55) |
| Vegetarians | 172 | 1.24 (1.03,1.49) | 149 | 1.24 (1.02,1.51) | 143 | 1.30 (1.06,1.60) | 96 | 1.15 (0.89,1.48) | 172 | 1.27 (1.05, 1.52) |
| Vegans | 41 | 2.25 (1.61,3.14) | 30 | 2.01 (1.37,2.95) | 35 | 2.33 (1.62,3.35) | 32 | 2.63 (1.78,3.89) | 41 | 2.35 (1.69, 3.28) |
| p-heterogeneity^c^ |  | <0.001 |  | 0.001 |  | <0.001 |  | <0.001 |  | <0.001 |
|  |  |  |  |  |  |  |  |  |  |  |
| **Leg fractures** |  |  |  |  |  |  |  |  |  |  |
| Meat eaters | 227 | Reference | 194 | Reference | 158 | Reference | 100 | Reference | 227 | Reference |
| Fish eaters | 48 | 1.07 (0.77,1.48) | 39 | 1.05 (0.74,1.51) | 41 | 1.18 (0.82,1.69) | 23 | 0.94 (0.59,1.50) | 48 | 1.09 (0.79, 1.51) |
| Vegetarians | 73 | 1.01 (0.76,1.35) | 58 | 1.02 (0.74,1.40) | 70 | 1.24 (0.91,1.69) | 57 | 1.43 (1.00,2.04) | 73 | 1.03 (0.77, 1.37) |
| Vegans | 18 | 2.07 (1.24,3.45) | 11 | 1.69 (0.90,3.18) | 15 | 2.18 (1.25,3.82) | 13 | 2.61 (1.41,4.82) | 18 | 2.05 (1.23, 3.40) |
| p-heterogeneity^c^ |  | 0.042 |  | 0.44 |  | 0.047 |  | 0.007 |  | 0.047 |
|  |  |  |  |  |  |  |  |  |  |  |
| **Ankle fractures** |  |  |  |  |  |  |  |  |  |  |
| Meat eaters | 332 | Reference | 264 | Reference | 229 | Reference | 187 | Reference | 332 | Reference |
| Fish eaters | 58 | 0.83 (0.62,1.11) | 51 | 0.92 (0.67,1.26) | 50 | 0.90 (0.65,1.23) | 40 | 0.83 (0.59,1.19) | 58 | 0.84 (0.63, 1.13) |
| Vegetarians | 117 | 1.00 (0.79,1.26) | 96 | 1.09 (0.84,1.40) | 106 | 1.12 (0.87,1.44) | 93 | 1.11 (0.84,1.45) | 117 | 1.03 (0.82, 1.30) |
| Vegans | 13 | 1.00 (0.56,1.77) | 11 | 1.15 (0.62,2.13) | 11 | 1.08 (0.58,2.00) | 10 | 1.04 (0.54,2.01) | 13 | 1.05 (0.60, 1.86) |
| p-heterogeneity^c^ |  | 0.65 |  | 0.79 |  | 0.64 |  | 0.53 |  | 0.64 |
|  |  |  |  |  |  |  |  |  |  |  |
| **Other main site fractures**^d^ | |  |  |  |  |  |  |  |  |  |
| Meat eaters | 287 | Reference | 252 | Reference | 207 | Reference | 150 | Reference | 287 | Reference |
| Fish eaters | 57 | 0.99 (0.73,1.33) | 51 | 1.02 (0.75,1.40) | 50 | 1.06 (0.76,1.46) | 35 | 0.94 (0.64,1.38) | 57 | 0.99 (0.73, 1.33) |
| Vegetarians | 100 | 1.00 (0.78,1.29) | 82 | 0.96 (0.73,1.26) | 89 | 1.03 (0.78,1.36) | 71 | 1.00 (0.73,1.36) | 100 | 0.99 (0.77, 1.27) |
| Vegans | 23 | 1.64 (1.05,2.58) | 22 | 1.80 (1.13,2.87) | 19 | 1.51 (0.92,2.49) | 20 | 1.85 (1.12,3.07) | 23 | 1.59 (1.01, 2.49) |
| p-heterogeneity^c^ |  | 0.17 |  | 0.07 |  | 0.44 |  | 0.08 |  | 0.21 |

^a^ Limiting the analyses to participants with no disease history of diabetes, heart disease, stroke, or cancer at baseline.
^b^ All analyses were stratified by sex, method of recruitment (general practice or postal), and region (7 categories), and adjusted for year of recruitment (per year from ≤1994 to ≥1999), ethnicity (white, other, unknown), Townsend deprivation index (quartiles, unknown), education level (no qualifications, basic secondary (e.g. O level), higher secondary (e.g. A level), degree, unknown), physical activity (inactive, low activity, moderately active, very active, unknown), smoking (never, former, light, heavy, unknown), alcohol consumption (<1g, 1-7g, 8-15g, 16+ g/day), dietary supplement use (no, yes, unknown), height (5cm categories from <155 to ≥185cm, unknown), BMI (<18.5, 18.5-19.9, 20-22.4, 22.5-24.9, 25-27.4, 27.5-29.9, 30-32.4, ≥32.5 kg/m^2^, unknown), and in women menopausal status (premenopausal, perimenopausal, postmenopausal, unknown), hormone replacement therapy use (never, ever, unknown), and parity (none, 1-2, ≥3, unknown). Total energy intake was adjusted for as sex-specific quintiles in the model with this additional adjustment.
^c^ Represents heterogeneity in risk between diet groups based on Wald tests.
^d^ Includes fractures of the clavicle, rib or vertebra.

**Table S6**: Risks of hip fractures by diet group, stratified by age, sex, menopausal status, physical activity and BMI.

| **Stratifying variable** | **N Cases in strata, hazard ratios (95% confidence intervals)**^a^ | | | | **Test of interaction**^b^ |
| --- | --- | --- | --- | --- | --- |
| **Age at recruitment** |  | **<50 years** |  | **≥50 years** |  |
| Meat eaters | 56 | Reference | 554 | Reference |  |
| Fish eaters | 20 | 1.25 (0.72,2.15) | 102 | 1.25 (1.00,1.55) |  |
| Vegetarians | 27 | 1.00 (0.61,1.66) | 145 | 1.31 (1.07,1.59) | χ^2^=4.96 |
| Vegans | 9 | 2.41 (1.12,5.19) | 32 | 2.27 (1.56,3.29) | p=0.29 |
| p-heterogeneity^c^ |  | 0.12 |  | <0.001 |  |
|  |  |  |  |  |  |
| **Sex** |  | **Men** |  | **Women** |  |
| Meat eaters | 103 | Reference | 507 | Reference |  |
| Fish eaters | 14 | 1.02 (0.57,1.83) | 108 | 1.30 (1.04,1.61) |  |
| Vegetarians | 34 | 1.20 (0.78,1.85) | 138 | 1.26 (1.03,1.54) | χ^2^=0.61 |
| Vegans | 10 | 1.77 (0.88,3.59) | 31 | 2.49 (1.70,3.63) | p=0.90 |
| p-heterogeneity^c^ |  | 0.42 |  | <0.001 |  |
|  |  |  |  |  |  |
| **Menopausal status**^d^ |  | **Premenopausal** |  | **Postmenopausal** |  |
| Meat eaters | 5 | Reference | 462 | Reference |  |
| Fish eaters | 3 | 0.93 (0.20,4.24) | 93 | 1.31 (1.04,1.65) |  |
| Vegetarians | 4 | 0.66 (0.17,2.63) | 118 | 1.31 (1.05,1.62) | χ^2^=0.85 |
| Vegans | 1 | 1.65 (0.17,16.40) | 25 | 2.41 (1.58,3.66) | p=0.93 |
| p-heterogeneity^c^ |  | 0.87 |  | <0.001 |  |
|  |  |  |  |  |  |
| **Physical activity** |  | **Inactive/low** |  | **Moderate/high** |  |
| Meat eaters | 407 | Reference | 99 | Reference |  |
| Fish eaters | 71 | 1.15 (0.88,1.49) | 27 | 1.33 (0.84,2.09) |  |
| Vegetarians | 114 | 1.23 (0.98,1.54) | 31 | 1.04 (0.67,1.61) | χ^2^=0.40 |
| Vegans | 25 | 2.16 (1.42,3.30) | 9 | 1.91 (0.92,3.98) | p=0.98 |
| p-heterogeneity^c^ |  | 0.003 |  | 0.26 |  |
|  |  |  |  |  |  |
| **Body mass index** |  | **<22.5 kg/m^2^** |  | **≥22.5 kg/m^2^** |  |
| Meat eaters | 209 | Reference | 357 | Reference |  |
| Fish eaters | 60 | 1.20 (0.89,1.62) | 57 | 1.38 (1.03,1.85) |  |
| Vegetarians | 87 | 1.21 (0.93,1.59) | 78 | 1.48 (1.14,1.92) | χ^2^=10.0 |
| Vegans | 33 | 3.17 (2.13,4.71) | 5 | 0.94 (0.38,2.29) | p=0.041 |
| p-heterogeneity^c^ |  | <0.001 |  | 0.01 |  |

^a^ Results shown were for subset analyses by the stratifying variable. Analyses were stratified by sex, method of recruitment (general practice or postal), and region (7 categories), and adjusted for year of recruitment (per year from ≤1994 to ≥1999), ethnicity (white, other, unknown), Townsend deprivation index (quartiles, unknown), education level (no qualifications, basic secondary (e.g. O level), higher secondary (e.g. A level), degree, unknown), physical activity (inactive, low activity, moderately active, very active, unknown), smoking (never, former, light, heavy, unknown), alcohol consumption (<1g, 1-7g, 8-15g, 16+ g/day), dietary supplement use (no, yes, unknown), height (5cm categories from <155 to ≥185cm, unknown), BMI (<18.5, 18.5-19.9, 20-22.4, 22.5-24.9, 25-27.4, 27.5-29.9, 30-32.4, ≥32.5 kg/m^2^, unknown), and in women menopausal status (premenopausal, perimenopausal, postmenopausal, unknown), hormone replacement therapy use (never, ever, unknown), and parity (none, 1-2, ≥3, unknown), except the stratifying variable where appropriate.
^b^ Interactions by age at recruitment, sex, menopausal status, physical activity and body mass index were investigated by including both strata in the model (e.g. both men and women) and comparing Cox models with and without the appropriate interaction term using likelihood ratio tests.
^c^ Represents heterogeneity in risk between diet groups based on Wald tests.
^d^ Premenopausal women included women who were below age 50 years at recruitment if they were perimenopausal or had unknown menopausal status, analyses in premenopausal women were censored at age 50. Postmenopausal women included women above age 50 years at recruitment if perimenopausal or had unknown perimenopausal status.
